# Supplementary material for: Armadillo-repeat kinesin1 interacts with Arabidopsis atlastin RHD3 to move ER with plus-end of microtubules
Source: Nat Commun. 2020 Nov 2;11:5510. doi: 10.1038/s41467-020-19343-2 (PMC7606470; doi:10.1038/s41467-020-19343-2)
Supplement: Supplementary file 1 — Supplementary Information [file 41467_2020_19343_MOESM1_ESM.pdf]

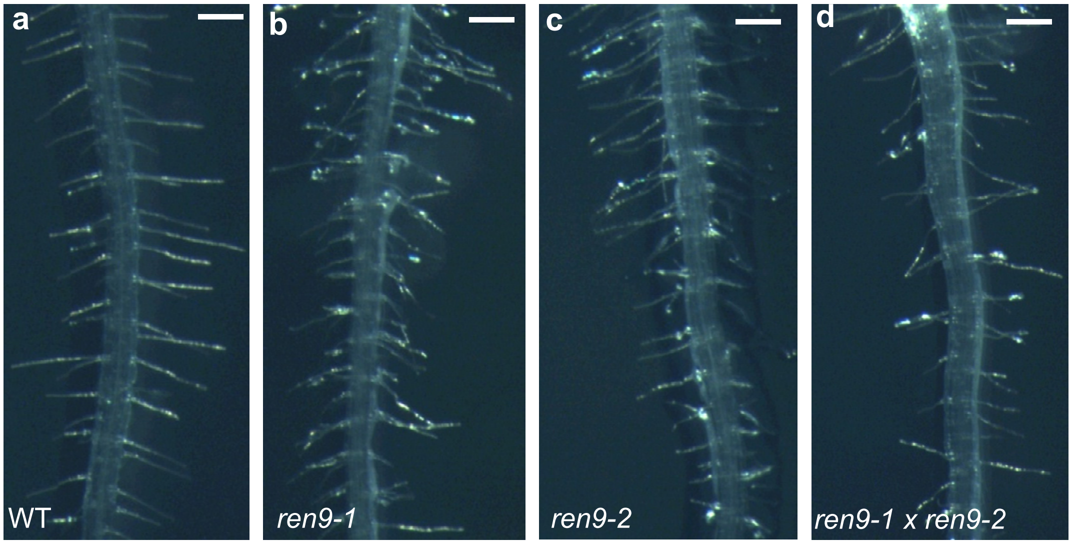

Supplementary Figure 1. *ren9-1* and *ren9-2* are alleles. The root hair phenotype of *ren9-2* (**c**) is identical to *ren9-1* (**b**). the genetic cross between the two alleles (**d**) indicates that the two alleles do not complement each other. WT – wild type. Scale Bars = 0.5mm

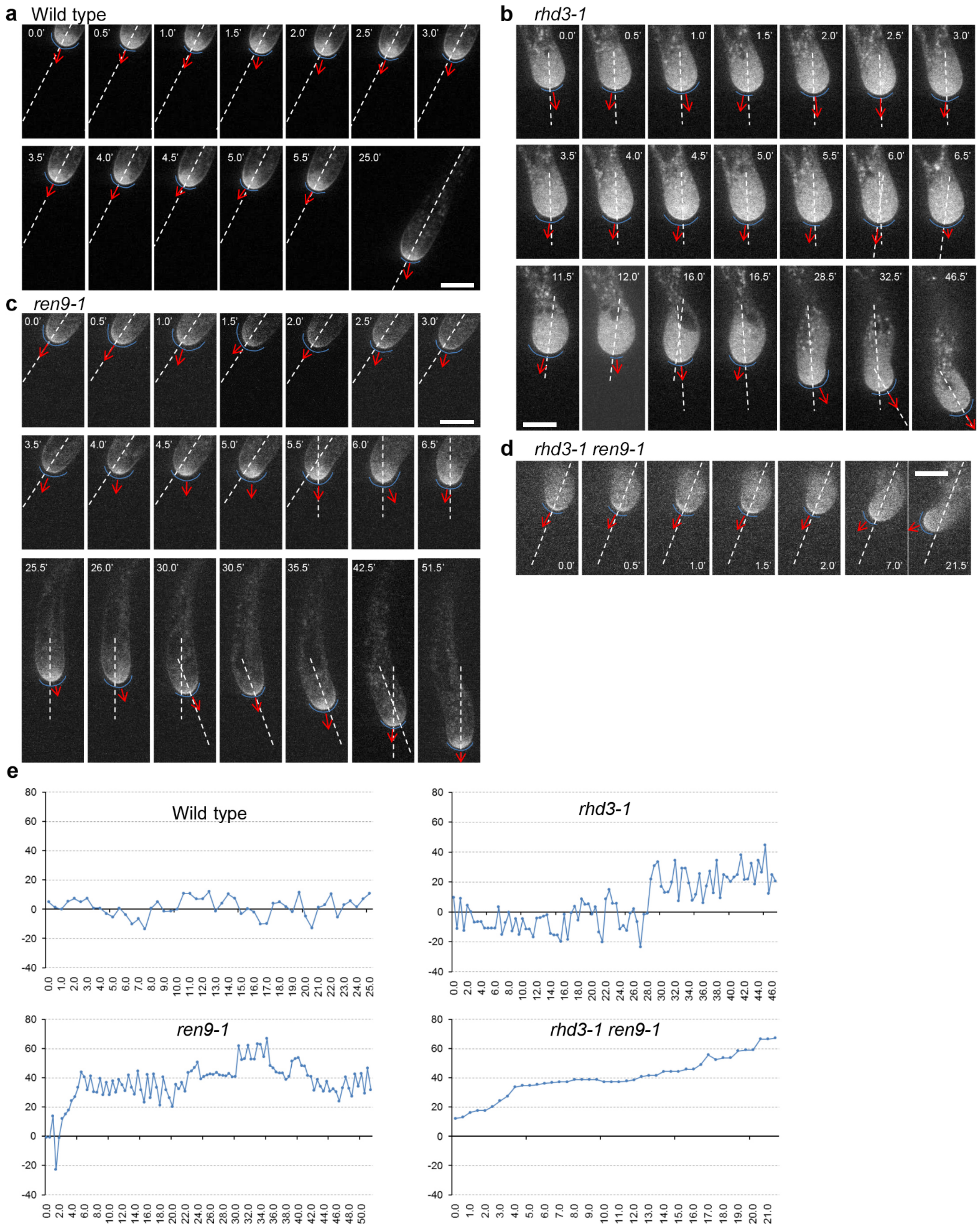

Supplementary Figure 2. Targeting of YFP-CLSD3 at the tips of growing root hairs. (**a-d**) Targeting of YFP-CLSD3 in growing root hairs of wild-type (a), *rhd3-1* (b), *ren9-1* (c), and *rhd3-1 ren9-1* (d). (**e**) Quantitative swing degrees by targeting of YFP-CLSD3 relative to the previous direction of root hair growth (indicated by white dashed lines). Root hairs expressing YFP-CLSD3 were imaged under spinning disc confocal microscopy. Images at the midplane are shown. The experiment was repeated 6 times. Scale bars = 10 μm. Blue lines indicate the focused localization of YFP-CLSD3 at apical plasma membrane. Arrows indicate targeting of YFP-CLSD3. White dashed lines present previous direction of root-hair growth. Swing suspensions were seen in 28.5'-46.5' in *rhd3-1*; 3.0'-6.0' and 25.5'-30' in *ren9-1*; and the swing was totally abolished in *rhd3-1 ren9-1*.

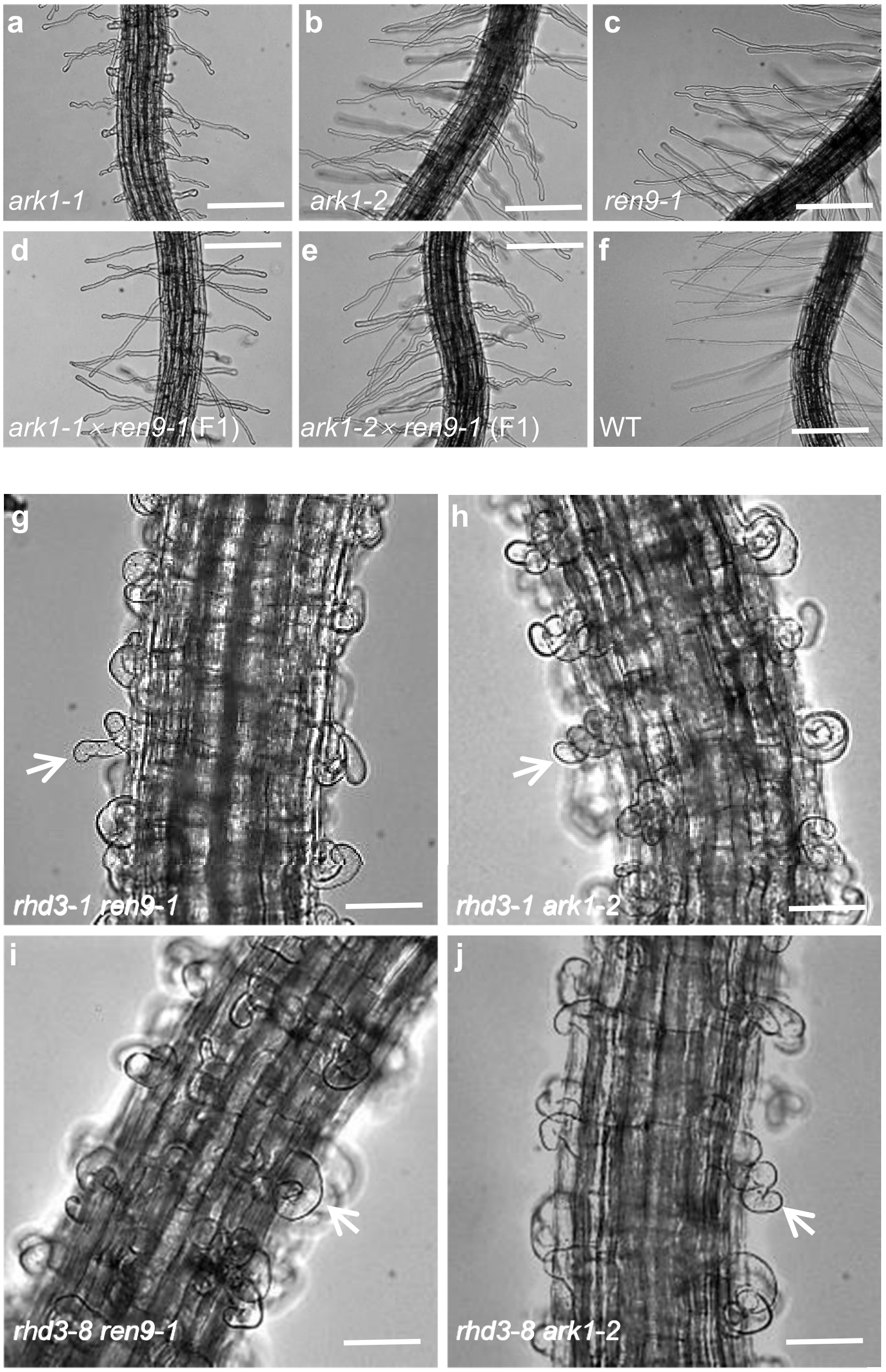

Supplementary Figure 3. Genetic complementation tests between *ren9-1* and *ark1-1* or *ark1-2*. (**a-f**) *ren9-1* failed to complement *ark1-1* and *ark1-2*, respectively. Root segments of 5-day old seedlings of *ark1-1* (a), *ark1-2* (b), *ren9-1* (c), *ark1-1 ren9-1* (d), *ark1-2 ren9-1* (e) and wild type (f). Scale bars = 0.5mm. (**g-j**) Root hairs of *rhd3-1 ren9-1* (g), *rhd3-1 ark1-2* (h), *rhd3-8 ren9-1* (i), and *rhd3-8 ark1-2* (j). Scale bars = 50μm. Arrows indicate the typical phenotype of *rhd3-1 ren9-1*.

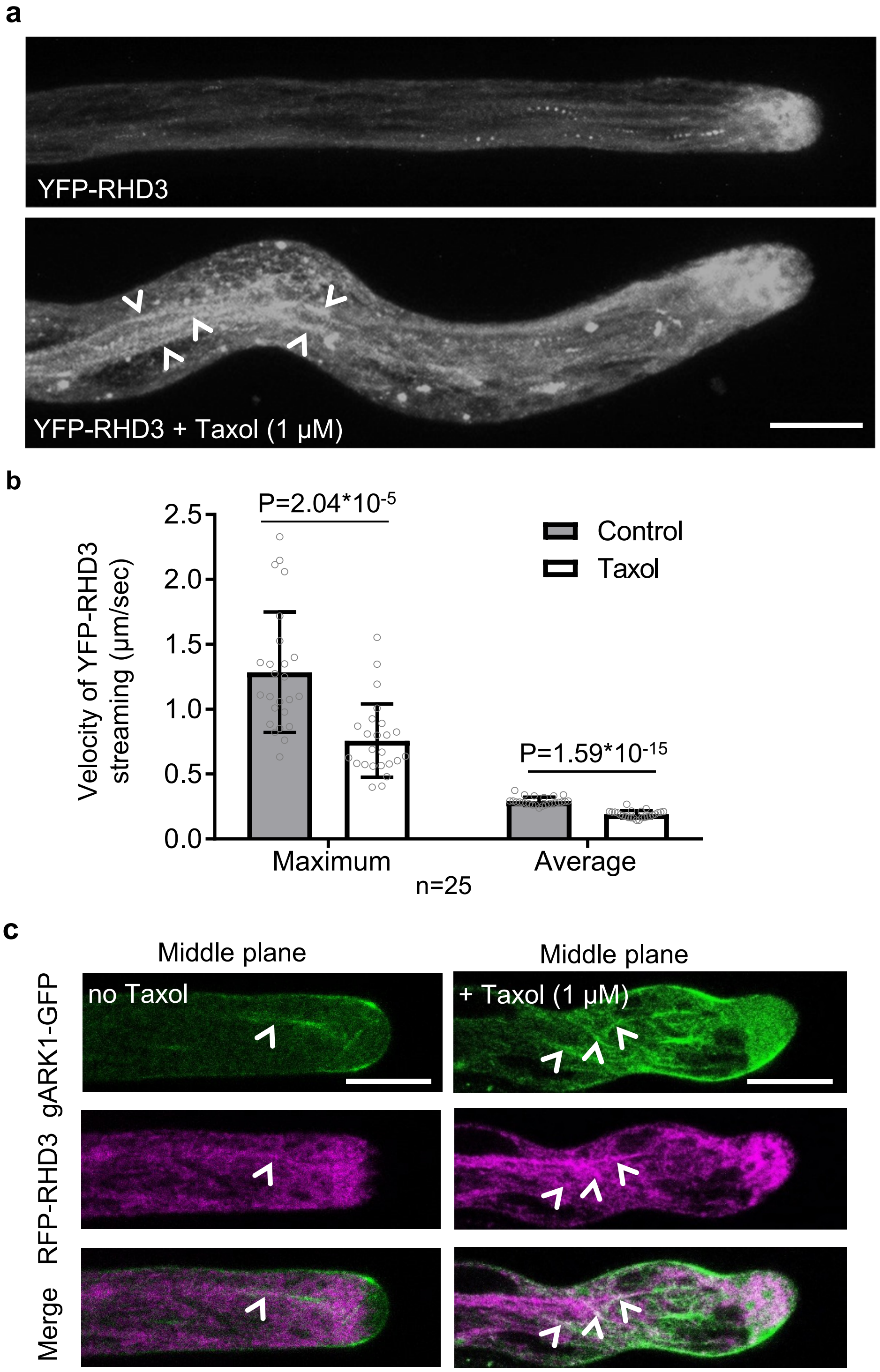

Supplementary Figure 4. Taxol (1μM) treatment mimics the effect of *ren9* mutations in distribution and motility of RHD3. (**a**) Distribution of YFP-RHD3 in growing root hairs of *rhd3-1* and *ren9-1*. Arrowheads indicate RHD3 bundles in the endoplasm. Scale bar = 10 μm. (**b**) The maximal and average velocity of YFP-RHD3 in taxol-treated root hairs and the control. Columns = mean, Error bars = standard deviation. Gray circles represent individual data value. p values indicated for significances are determined by Student's *t*-test (two-sided). n = individual root hairs. (**c**) Distribution of gARK1-GFP and RFP-RHD3 in the midplane of a root hair without (left panel) and with (right panel) taxol (1μM). Arrowheads indicate bundles and colocalization of gARK1-GFP and RFP-RHD3 in the endoplasm. The experiment was repeated 3 times. Scale bars = 10 μm. An increase in colocalization of gARK1-GFP and RFP-RHD3 was evident in root hairs treated with taxol (1μM).

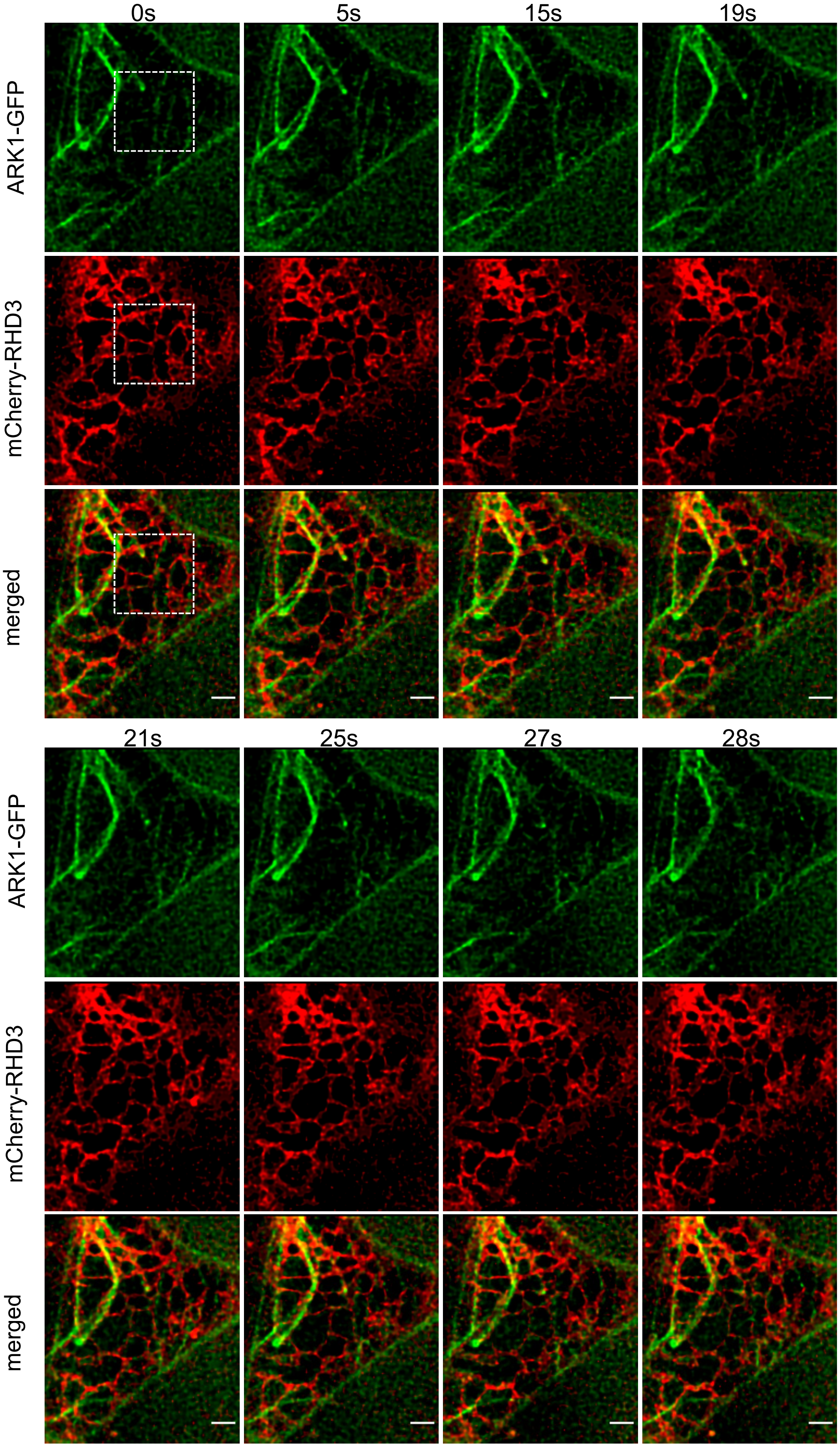

Supplementary Figure 5. A large field view of Figure 9a where ARK1-GFP co-moves with mCherry-RHD3 during the time. Scale bars = 2μm. The dashed boxes indicate the enlarged region showing in the Figure 9a. The experiment was repeated 9 times.

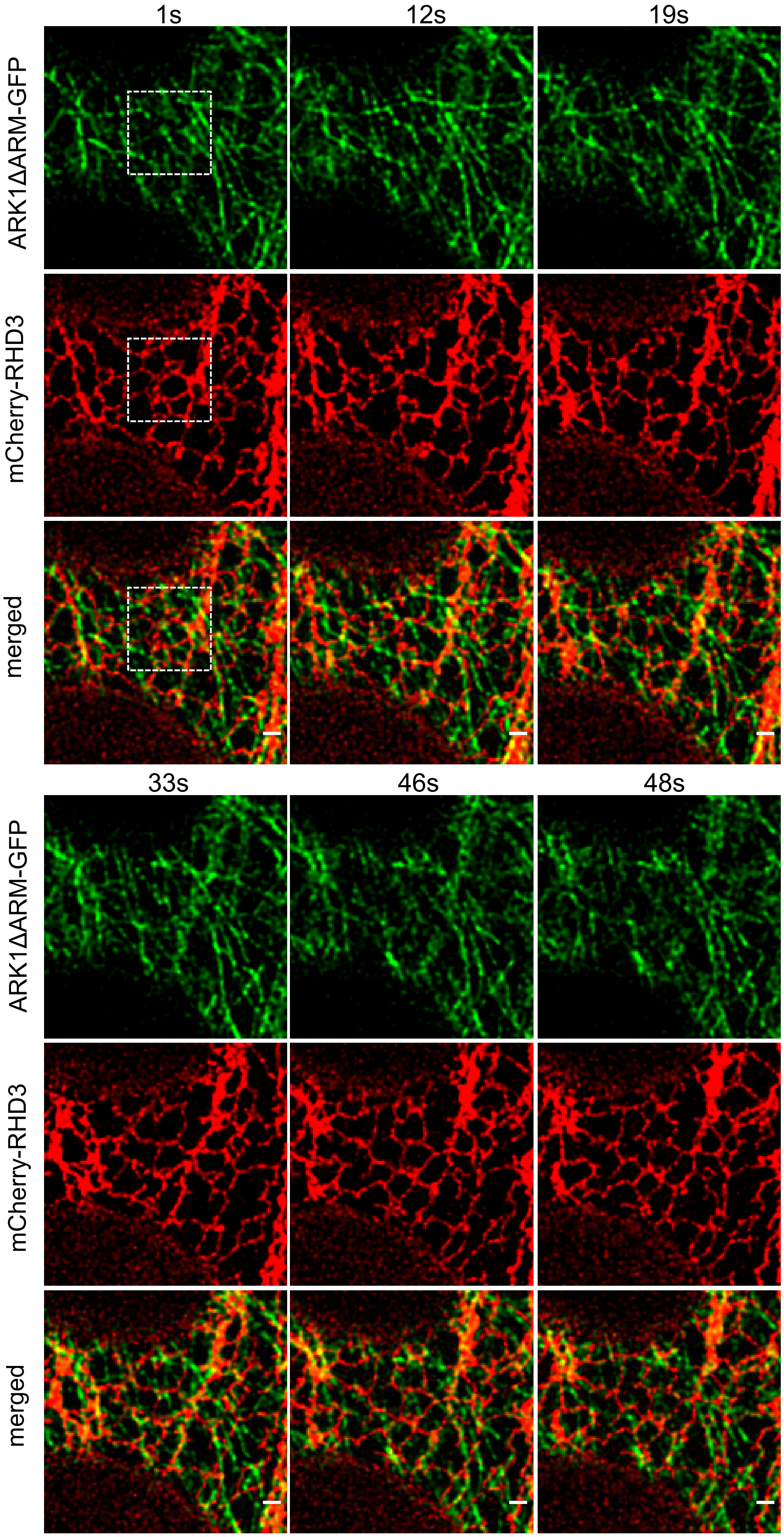

Supplementary Figure 6. A large field view of Figure 9c where ARK1ΔARM-GFP does not move with mCherry-RHD3 during the time. Scale bars = 2μm. The dashed boxes indicate the enlarged region showing in the Figure 9c. The experiment was repeated 4 times.
