# Supplementary material for: Armadillo-repeat kinesin1 interacts with Arabidopsis atlastin RHD3 to move ER with plus-end of microtubules
Source: Nat Commun. 2020 Nov 2;11:5510. doi: 10.1038/s41467-020-19343-2 (PMC7606470; doi:10.1038/s41467-020-19343-2)
Supplement: Supplementary file 3 — Descriptions of Additional Supplementary Files [file 41467_2020_19343_MOESM3_ESM.pdf]

Descriptions of Additional Supplementary Files

Supplementary Movie 1

Description: gARK1-GFP is localized largely to the growing plus end of MTs.

Supplementary Movie 2-4

Description: 3D modeling of a z-stack of a growing root hair expressing gARK1-GFP (movie 2 and green in merged movie 4) and mCherry-MAP4 (movie 3 and red in merged movie 4).

Supplementary Movie 5

Description: gARK1-GFP and RFP-RHD3 in the middle plane of the subapical region as the root hair elongated.

Supplementary Movie 6

Description: gARK1-GFP and with RFP-RHD3 in the cortex of the subapical region as the root hair elongated.

Supplementary Movie 7-9

Description: 3D modeling of a z-stack of a growing root hair expressing gARK1-GFP (movie 7 and green in merged movie 9) and RFP-RHD3 (movie 8 and red in merged movie 9).

Supplementary Movie 10

Description: Distribution of YFP-RHD3 in a growing root hair of rhd3-1.

Supplementary Movie 11

Description: Distribution of YFP-RHD3 in a growing root hair of ren9-1.

Supplementary Movie 12

Description: Directional movement of gAKR1-GFP in a growing root hair of ren9-1.

Supplementary Movie 13

Description: Randomized movement of gAKR1-GFP in a growing root hair of rhd3-1.

Supplementary Movie 14

Description: ARK1-GFP comet moves together with mCherry-RHD3 during the elongation of an ER tubule and after the formation of a 3-way ER junction.

Supplementary Movie 15

Description: ARK1ΔARM-GFP comet does not move together with mCherry-RHD3.
