# Supplementary material for: Armadillo-repeat kinesin1 interacts with Arabidopsis atlastin RHD3 to move ER with plus-end of microtubules
Source: Nat Commun. 2020 Nov 2;11:5510. doi: 10.1038/s41467-020-19343-2 (PMC7606470; doi:10.1038/s41467-020-19343-2)
Supplement: Supplementary file 19 — Reporting Summary [file 41467_2020_19343_MOESM19_ESM.pdf]

nature research

Corresponding author(s): Huanquan Zheng

Last updated by author(s): Oct 8, 2020

Reporting Summary

Nature Research wishes to improve the reproducibility of the work that we publish. This form provides structure for consistency and transparency in reporting. For further information on Nature Research policies, see our Editorial Policies and the Editorial Policy Checklist.

Statistics

For all statistical analyses, confirm that the following items are present in the figure legend, table legend, main text, or Methods section.

n/a	Confirmed	
☐	☒	The exact sample size (n) for each experimental group/condition, given as a discrete number and unit of measurement
☐	☒	A statement on whether measurements were taken from distinct samples or whether the same sample was measured repeatedly
☐	☒	The statistical test(s) used AND whether they are one- or two-sided<br>*Only common tests should be described solely by name; describe more complex techniques in the Methods section.*
☒	☐	A description of all covariates tested
☒	☐	A description of any assumptions or corrections, such as tests of normality and adjustment for multiple comparisons
☐	☒	A full description of the statistical parameters including central tendency (e.g. means) or other basic estimates (e.g. regression coefficient) AND variation (e.g. standard deviation) or associated estimates of uncertainty (e.g. confidence intervals)
☐	☒	For null hypothesis testing, the test statistic (e.g. F, t, r) with confidence intervals, effect sizes, degrees of freedom and P value noted<br>*Give P values as exact values whenever suitable.*
☒	☐	For Bayesian analysis, information on the choice of priors and Markov chain Monte Carlo settings
☒	☐	For hierarchical and complex designs, identification of the appropriate level for tests and full reporting of outcomes
☒	☐	Estimates of effect sizes (e.g. Cohen's d, Pearson's r), indicating how they were calculated

Our web collection on statistics for biologists contains articles on many of the points above.

Software and code

Policy information about availability of computer code

Data collection	Fiji version 1.42 (www.fiji.sc) and Huygens version 15.05. 5 (www.svi.nl)
Data analysis	following Fiji modules were used: segmented line, angle tool, KbiFlow, MTrackJ, following Huygens modules were used: the Surface renderer, the Deconvolution Express

For manuscripts utilizing custom algorithms or software that are central to the research but not yet described in published literature, software must be made available to editors and reviewers. We strongly encourage code deposition in a community repository (e.g. GitHub). See the Nature Research guidelines for submitting code & software for further information.

Data

Policy information about availability of data

All manuscripts must include a data availability statement. This statement should provide the following information, where applicable:

- Accession codes, unique identifiers, or web links for publicly available datasets
- A list of figures that have associated raw data
- A description of any restrictions on data availability

Accession codes for all genes studied have been provided, all figures have raw data, there is no restriction on data availability

Field-specific reporting

Life sciences study design

All studies must disclose on these points even when the disclosure is negative.

Sample size	1. To measure the length and growth bending angle of root hairs, around 100 samples were collected from 5 experimental replications.<br>2. To quantify the distribution of RHD3 in wild type and ren9-1, the apex length and sub-apex length was calculated from 25 YFP-RHD3 expressed individual root hairs of each sample. Root hairs were randomly selected from three experiments and imaged.<br>3. To quantify the velocity of RHD3 in wild type and ren9-1, 25 YFP-RHD3 expressed individual root hairs of each sample randomly selected from three experiments were imaged.<br>4. To quantify the velocity of gARK1-GFP in wild type and rhd3-1, 9-21 gARK1-GFP expressed individual root hairs of each sample randomly selected from three experiments were imaged.<br>5. To quantify the velocity of RHD3 in wild type +- taxol, YFP-RHD3 expressed individual root hairs were treated and 25 root hairs of non-treated and treated samples were randomly selected from three experiments were imaged.<br>The mean values, SD and SE were calculated, and student t-test were also conducted.
Data exclusions	all data are randomly collected, there was no data exclusions
Replication	at least 3 independent experimental replicates for each experiments were carried out
Randomization	all samples were randomly collected
Blinding	Blinding was not relevant in this study as this is a molecular biology based analysis of some plant samples. Two post-docs , a PhD student and an MSc student were involved in their respective data collections

Reporting for specific materials, systems and methods

We require information from authors about some types of materials, experimental systems and methods used in many studies. Here, indicate whether each material, system or method listed is relevant to your study. If you are not sure if a list item applies to your research, read the appropriate section before selecting a response.

Materials & experimental systems

n/a	Involved in the study	
☐	☒	Antibodies
☒	☐	Eukaryotic cell lines
☒	☐	Palaeontology and archaeology
☒	☐	Animals and other organisms
☒	☐	Human research participants
☒	☐	Clinical data
☒	☐	Dual use research of concern

Methods

n/a	Involved in the study	
☒	☐	ChIP-seq
☒	☐	Flow cytometry
☒	☐	MRI-based neuroimaging

Antibodies

Antibodies used	anti-GFP (Abcam, ab32146), anti-mCherry ((Abcam, ab34771), anti-rabbit IgG-peroxidase (Sigma A4914-1ML)
Validation	These two antibodies are commercial available and are widely used in the field. There are many publications that used these two antibody.
